# Comparison of osseodensification and standard drilling methods on implant stability quotient and insertion torque values of implants

**DOI:** 10.34172/japid.025.3475

**Published:** 2025-04-23

**Authors:** Omid Moghaddas, Vanda Banazadeh

**Affiliations:** Private Practice, Tehran, Iran

**Keywords:** Insertion torque, Osseodensification, Standard drilling

## Abstract

**Background.:**

This study aimed to compare insertion torque (IT) and implant stability quotient (ISQ) values immediately and three months after surgery with standard drilling (SD) and osseodensification (OD) drilling methods.

**Methods.:**

In this prospective study, 39 implants with the same diameter and length were placed in 21 patients using SD (23 implants) and OD (16 implants) methods in the posterior maxilla. The amounts of IT after surgery and ISQ after surgery and three months later were evaluated. The data were extracted and statistically analyzed with PASS2 software. The difference between IT and ISQ was determined by the two-way repeated-measures ANOVA at a significance level of 0.001.

**Results.:**

Immediately after surgery, the amount of IT using the OD drilling method was 37% higher than the SD drilling (*P*<0.001). ISQ values after surgery did not show a significant difference between SD and OD (*P*1<0.176). Three months after surgery, the ISQ values in both groups were not significantly different. ISQ values for all OD and SD group experimental parameters remained above the threshold value of 68. IT values showed a positive correlation with ISQ values at baseline.

**Conclusion.:**

In the present study, the OD technique provided a higher IT rate after surgery compared to the SD method; however, no difference was seen between the two groups regarding ISQ value either after surgery or three months later.

## Introduction

 Osseointegration is the formation of a structural and functional bone-to-implant interface, without soft tissue interference, where bone metabolism is challenged by a foreign body that induces stress/strain in the peri-implant tissue and triggers highly integrated and complex immunomodulated inflammatory reactions, eventually leading to new bone formation.^1,2^

 Achieving the best degree of primary stability during surgery, defined as bone-to-implant biomechanical engagement with a micromotion < 150 μm, is necessary for successful osseointegration and to predict loading time.^3,4^ Clinically, the degree of implant stability can be estimated by the insertion torque (IT) values using surgical handpieces or obtaining implant stability quotients (ISQ) using resonance frequency analysis. IT values > 35 Ncm or ISQ values > 68 have been considered reasonable for predictable osseointegration and earlier loading.^5^ These values should be achieved after implant placement and maintained over the initial course of osseointegration.

 According to bone elastic properties, there is a linear relationship between the interfacial stress distribution during implant installation and the respective peri-implant tissue strain due to frictional forces. Therefore, bone density in the peri-implant vicinity, implant macro-geometry, and related surgical instrumentation are critical morphometric predictors of IT and healing kinetics.^6,7^

 A series of studies have investigated the relationship between such factors, where the mismatch between the implant and the bone walls around it dictates the course of osseointegration around the metallic device through predominantly interfacial bone remodeling, predominantly intramembranous-like healing, or hybrid healing pathways, which affects the rate at which secondary stability occurs.^8,9^

 Some of the newest tools introduced are counterclockwise drills that are used to increase bone density, which is currently known as osseodensification (OD). In the clockwise direction, they also have the efficiency of conventional drills. OD drills include a non-subtractive method that increases bone density while expanding the implant site.^10^

 The relatively new OD osteotomy preparation technique has prompted a paradigm shift in implant site preparation through a multi-step drilling concept using uniquely designed burs that cause the lateralization of autogenous bone into the surrounding cancellous structure and expand the surrounding bone space by rolling and sliding contact with controlled bone deformation, all with minimal heat elevation.^10,11^

 This OD technique is based on bone’s elastic and plastic properties to preserve bone and its density. As a result, grafting autograft bone material to the trabecular space and increasing its density may be especially useful in clinical scenarios with low bone quality.

 This OD^10^ technique is based on the bone elastic and plastic properties, which causes bone bulk preservation and compaction, resulting in the autografting of an osseous material into the trabecular space and increasing its density, which might be especially useful in clinical scenarios with poor bone quality.^11,12^

 Preclinical biomechanical and histological data have shown significantly higher IT and temporal removal torque for OD compared to standard conventional drilling (SD).^10,13,14^ According to the results of torque removal measurements during the healing period, preparation using the OD method is suggested due to biomechanical advantages.^13^

 Therefore, this study compared OD and SD methods on the implant’s insertion torque (IT) and ISQ.

## Methods

###  Study design

 This study was designed as a prospective evaluation to investigate the influence of two osteotomy techniques, OD (test group) and SD (control group), on clinical implant osseointegration parameters after the surgery and three months later, according to CONSORT guidelines.

 The procedures followed the ethical standards of the responsible committee of human experimentation (institutional and national) and with the Helsinki Declaration of 1975, as revised in 2008.

 Since osteotomy (SD and OD) were the independent variables evaluated, the minimum sample size was calculated with a two-sample t-test (PASS2) to obtain a 5% α error and 20% β error within an effect size of 18 at n = 8 implants, which was increased by 20% to account for potential losses and refusals. Approximately 20 implants were required per factor (20 OD, 20 SD), totaling at least 40 implants.

 Choosing a surgical technique that improves implant stability is very important to achieve successful osseointegration, especially in areas where bone quality is compromised, such as the posterior region of the maxilla; hence, techniques to achieve predictable primary stability are needed. OD is among the recent well-evaluated techniques for achieving primary dental implant stability. This study used the original kit by Versah Company (USA) for site preparations.

 Patients needing dental implants in the posterior maxilla were recruited from 2021 to 2022. All the patients were subjected to a preliminary evaluation that included a careful review of their medical and dental histories, detailed clinical examination, and evaluation of oral hygiene. Inclusion criteria included patients at least 18 years of age, sufficient residual bone volume for implant placement without needing bone augmentation, where the minimum ridge height and width should be ≥ 9 and ≥ 6 mm, respectively, healed sites with at least 4 months of post-extraction period. The exclusion criteria were alcoholism, smoking, use of illicit drugs, heart diseases, diabetes, previous bone regenerative or augmentation procedures, bleeding disorders, compromised immune system, irradiated patients, and previous or active treatment with steroids or bisphosphonates.

 All the patients underwent radiographic evaluations, including periapical radiographs and cone-beam computerized tomography scans before implant placement for surgical planning and assessment of bone dimensions around the implantation site. The implant system utilized had an internal conical connection, tapered macro-geometry, and a sandblasted surface: MEGAGEN ANYONE (South Korea).

 Each patient received a detailed description of the study protocol, and after completing the written consent form, the population was studied.

 The operator performing the IT and ISQ readings was blinded as to the drilling protocol used.

###  Surgical technique

 The patients were instructed to rinse with 0.2% chlorhexidine solution for 1 minute and expectorate. After these preoperative procedures were completed, sterile surgical drapes were used to cover the patient’s chest to minimize the potential contamination from extraoral sources. The surgical procedure was performed under local anesthesia (lidocaine with 1:100 000 epinephrine). After local anesthesia was achieved, full‐thickness surgical flaps were elevated, and implant osteotomies were performed with the assistance of saline irrigation. The osteotomies were performed at 1100 rpm using sequential burs of similar diameter for both surgical techniques (SD conventional burs or OD drilling burs) and with a maximum torque of 50 Ncm. The instrumentation was performed according to the recommended drilling protocols for each implant system, either by SD, as recommended by specific implant company protocols, or by OD, as recommended by the densifying reference guide for each specific implant system. The insertion of the implants was initiated with the motor handpiece, without irrigation, at 20–50 rpm. Installation was completed with a manual surgical torque wrench indicator. In both the case and control groups, the implants were placed 1 mm subcrestal, and their diameter and length were 4 × 10. IT values were recorded as the maximum torque value (Ncm) reached the termination of implant insertion.

 After the final seating of the implant, a Smartpeg specific for the implant system and restorative platform diameter was used for each implant, and a resonance frequency analysis was performed using an MEGA ISQ (Megagen, South Korea) to record ISQ values in all implant surfaces. New, sterile healing abutments were inserted after the implant installation, and the incision was sutured to close the wounds. These sutures were removed 10 days postoperatively.

 ISQ values were also recorded after 3 months of healing during follow‐up visits. Healing abutments were placed after surgery so subsequent ISQ readings could be readily obtained. After healing, an impression of the implant’s spatial positioning and orientation was made, and the final restoration was fabricated according to the respective clinical scenario.

 All the patients were instructed to follow a soft and tepid diet in the first three days after surgery, along with instructions for oral hygiene. They received a prescription for amoxicillin (500 mg, one tablet every 8 hours for 7 days), starting 1 hour before surgery. The rationale for this sequencing of measurements comes from derived curves of primary versus secondary stability development, suggesting that decreased stability is generally expected 3–5 weeks after implantation.

###  Statistical analysis

 In this research, after data collection, two-way repeated-measures ANOVA was used to calculate IT and ISQ in two groups at different times due to the data’s normal distribution.

 This study was designed as a prospective evaluation and investigated the effect of the osteotomy technique at the time of implant placement and three months later on the clinical parameters of osseointegration, according to CONSORT guidelines.

## Results

 Twenty-one patients, including 10 women and 11 men, with an average age of 40 years, meeting the inclusion criteria, participated in this study. In total, 39 implants were placed in the posterior maxilla, including 18 OD implants and 20 SD implants with the same diameter (4 mm).

 In general, the final healing of the implants proceeded without any signs of inflammation or infection of the tissues around the implant and the mobility of the implant in the second stage of surgery.

 Twenty implants in each group were statistically analyzed. In follow-ups, two patients (two implants) could not cooperate and were excluded from the study.

 The results showed that at the time of surgery, the amount of IT by the OD drilling was 37% higher than the SD drilling (50.31 ± 5.90 Ncm vs. 36.09 ± 11.07 Ncm) (*P* < 0.001), which was statistically significant (*P* < 0.001).

 ISQ values at the time of surgery did not show any significant difference between the SD and OD groups (76.13 ± 6.07 vs. 71.74 ± 2.42) (*P*1 < 0.134 and *P*1 < 0.176). After three months, ISQ values in both groups did not change much or show a significant difference. (75.13 ± 6.44 vs. 73.43 ± 1.67) (*P*4 < 0.488 and *P*4 < 0.471) (Table 1). ISQ values for OD and SD groups were high at baseline and remained high continuously. ISQ values for all OD and SD experimental parameters remained above the threshold value of 68. IT values showed a positive correlation with ISQ values at zero time.

**Table 1:** Average values of ISQ (implant stability quotient) immediately after surgery and three months later with OD and SD methods

**Group type/** ***P*** ** value**	**After surgery**	**Three months later**
OD group (n = 18)	76.13 ± 6.44	75.13 ± 6.44
SD group (n = 20)	71.74 ± 6.44	73.43 ± 6.4
*P* value	*P*1 < 0.134	*P*4 < 0.471

## Discussion

 Selecting surgical instrumentation which improves implant stability in the alveolar bone is necessary for successful osseointegration.^7,8,14,15^ Historically, curves of primary versus secondary stability have suggested decreased stability 2–4 weeks after surgery.^16^

 An alternative approach has been proposed and developed to overcome the limitations of the standard method. Instead of removing bone particles in conventional SD techniques, it has been proposed that an OD drilling sequence preserves bone by compacting the particles into the osteotomy wall.^10,11^ Studies have indicated significantly higher biomechanical and histomorphometric parameters for the OD method compared to the conventional SD method in temporal investigations.^10,13,17^

 This clinical trial investigated the effect of SD and OD methods on IT and ISQ immediately after surgery and three months later in implants placed in the posterior maxilla. IT and ISQ are two clinically accepted parameters to determine implant primary stability, where both higher IT and ISQ values are positive indicators for implant stability, which can be necessary for immediate loading and osseointegration. ISQ is an efficient indicator that compares subsequent measurements at set time intervals. This objective measure is independent and incomparable to the IT value obtained during surgery.

 This study showed that the OD technique increases the primary stability and bone volume on the implant surface by creating a layer of mineralized bone around the osteotomy site, which was also reported by Bergamo et al.^18^

 Data analysis showed higher IT values in OD than in SD. Successful osteotomy means that the implant is placed in a three-dimensional position with appropriate biomechanical stability, is prepared with accurate measurements and a series of drills, and is protected from damage caused by overheating.^7^ Achieving high levels of biomechanical stability is strongly required in clinical practice to accommodate the current tendency toward early loading protocols, especially for low-density bone types.^19,20^

 OD preserves the bone in two ways: densifying cancellous bone with viscoelastic and plastic deformation and autograft densification of bone particles along the length and apex of the osteotomy.^10^

 Therefore, the OD method has been demonstrated to improve bone quality as osteotomy size is expanded and guarantees greater levels of physical interlocking at the implant interface.^10,13,17,21,22^

 The maximized biomechanical behavior of the OD method is the use of specially designed drills for osteotomy, which use bone elastic and plastic properties while applying time-dependent stresses (forces) to create a time-dependent strain (deformation), compacting bone particles into the trabecular space instead of removing them.^10,11^ Such a technique has shown a sealing/bridging into the intra-thread spaces as a result of the reversed compression exerted by the bone spring-back effect created by the residual elastic strain generated during the osteotomy, without the excessive stress that would lead to extensive remodeling and decreased stability of conventional press-fit undersized preparations.^10,12,18^

 Counterclockwise rotation of the drill causes the drill to slide on the bone surface with less force than the ultimate strength of the bone. The interfacial stress distribution and the peri-implant tissue strain, due to frictional forces resulting from the interplay between osteotomy and macro geometry during implant placement, have been shown to control the mechanical interlocking necessary for increased primary stability and bone healing response.^7,8,14,15^ Bone tissue tolerates certain levels of compressive strain, even beyond the yield point, without affecting the osseointegration, which, through the elastic behavior, improves the physical engagement, resulting in higher IT and ISQ values. However, when the strain level is markedly higher than the yield point, the plastic deformation and the presence of microcracks may trigger extensive interfacial bone remodeling and decrease primary stability.^7,8,14,15,23^

 The implant’s stability depends on the direct contact between the surface of the implant and the bone so that micromotion is reduced at this interface. If a microfracture occurs, bone regeneration may take three months or more to repair damaged bone.

 Historically, using the SD method, the wider the osteotomy, the greater the amount of extracted bone, which creates an increased strain level generated by the interplay between implant and bone. This may result in more bone remodeling healing.^7,23,24^

 OD provides a balance between maintaining bone volume and higher implant stability due to the spring-back effect without creating a “misfit” and undersized osteotomies.^25^

 Based on the review study by Tretto et al,^25^ who compared the SD technique with OD, OT (osteotome), PD (piezoelectric), and LS (laser), SD was not different from other techniques in terms of bone-to-implant contact, but the OD technique was higher in IT, RT (removal torque), and ISQ. Unlike the present study, there was no significant difference in ISQ. All the biomechanical assessments showed significant benefits in the OD group, including higher RT and IT and increased primary and secondary stability. In one study, peri-implant bone volume was significantly higher in the OD group.

 Also, in Bergamo and colleagues’^18^ study, OD compared to SD increased the primary stability of the implant and the bone density. Unlike the present study, ISQ increased significantly in OD compared to SD. This clinical study on 56 patients examined variables such as mandible or maxilla, anterior or posterior, jaw, and implant size.

 The animal study of Huwais and Meyer^10^ investigated the effect of OD on primary stability, bone density, and bone-to-implant contact. As in the present study, IT increased significantly (twice) in the OD technique compared to SD, and no significant differences in ISQ were shown between the three groups. Although the same drill was used for extraction and OD drilling, the osteotomy diameters of OD were smaller than the other two techniques due to the spring-back effect. The percentage of bone around the OD group was almost three times that of the standard technique.

 An animal study by Lahens et al^24^ investigated the effect of OD on primary stability and osseointegration of endosteal implants in low-density bone: SD, clockwise (CW) OD, and counterclockwise (CCW) OD with Densah. As in the present study, IT showed higher values for OD (100 Ncm) than SD (25 Ncm). Also, the bone-to-implant contact was significantly higher (BIC). As a result, endosteal implants show higher IT in bone with low density when placed in OD drilling sites. Unlike the present study, ISQ was not investigated.

 Mello-Machado et al^16^ compared implant stability in low-quality bone by OD and SD techniques. Sixteen individuals with D3 or D4 bone density were randomly distributed to receive implants. IT and ISQ were measured immediately after implant placement. ISQ was evaluated after six months. As in the present study, the OD group showed higher IT than SD. ISQ values were similar to those in the present study. After six months, implant survival was similar in both groups, and ISQ values were higher. Unlike the present study, ISQ remained stable after three months.

 In an animal study, Trisi et al^26^ evaluated the OD technique to prepare the implant site; 10 implants were placed using the SD method, and 10 were placed using the OD method. No failures were observed after two months. A significant increase in ridge width and percentage of bone volume (BV percentage) (approximately 30% more) was observed in the OD group. Significantly better RT values were recorded for the OD group than for the SD group. The present in vivo study showed that it could increase the percentage of BV around implants placed in low-density bone compared to the SD technique, which might increase implant stability and reduce micromotion. Unlike the present study, IT and ISQ were not measured. Pai et al^27^ conducted a systematic review to analyze whether the OD method has advantages over conventional osteotomy regarding bone density and primary stability. A total of 195 articles were collected and screened using inclusion and exclusion criteria. It was observed that the use of Densah burs for OD caused undersized osteotomy compared to conventional burs. It also improved bone density and increased the percentage of bone volume and bone-to-implant contact, improving implant stability.

 According to the results, the current study also had limitations that must be considered for future studies. In this study, only the posterior maxilla was evaluated, and the anterior region was not considered. Other limitations of the present research include the number of patients. Even though the current data showed that OD improved the rapid performance of SD in accelerating osseointegration and improving primary and secondary stability, long-term prospective studies are needed to evaluate implant stability in different clinical scenarios in different types of bone and different sizes of micro- and macro-geometry implants.

## Conclusion

 The results showed that OD by maintaining bone structure and volume and increasing bone density was effective in improving and increasing the stability of the implant compared to the SD method, making it possible for the implant to be passively placed in the prepared site without causing stress despite its high stability.

## Competing Interests

 The authors declare that they have no competing interests.

## Data Availability Statement

 Detailed data of this study is available.

## Ethical Approval

 This study was approved by the ethical committee of Islamic Azad University, Dental Branch, Tehran under approval ID: IR.IAU.DENTAL.REC.1401.1402.
